# Supplementary material for: The fractal structure of communities of practice: Implications for business organization
Source: PLoS One. 2020 Apr 29;15(4):e0232204. doi: 10.1371/journal.pone.0232204 (PMC7190158; doi:10.1371/journal.pone.0232204)
Supplement: Attachment — Submitted filename: Responses to reviewers comments.docx [file pone.0232204.s002.docx]

PONE-D-19-29833
The Fractal Structure of Communities of Practice
PLOS ONE

Thank you for the opportunity to revise our paper in the light of the reviewers’ comments. We have revised the paper extensively in the light of these comments, as should be clear from the TRACK CHANGED version.

1. We have amended the title.
2. The data are provided in a Supplementary Materials file.
3. A new figure has been added and the figures have been restructured to support the text better.
4. The Discussion has been extended to emphasise the implications for business organisation.

Journal Requirements:

When submitting your revision, we need you to address these additional requirements.

1. Please ensure that your manuscript meets PLOS ONE's style requirements, including those for file naming. The PLOS ONE style templates can be found at

http://www.journals.plos.org/plosone/s/file?id=wjVg/PLOSOne_formatting_sample_main_body.pdf and http://www.journals.plos.org/plosone/s/file?id=ba62/PLOSOne_formatting_sample_title_authors_affiliations.pdf

2. Please consider changing the title so as to meet our title format requirement (https://journals.plos.org/plosone/s/submission-guidelines). In particular, the title should be "Specific, descriptive, concise, and comprehensible to readers outside the field" and in this case it is not informative and specific about your study's scope and methodology.

 Title amended

3. In your Data Availability statement, you have not specified where the minimal data set underlying the results described in your manuscript can be found. PLOS defines a study's minimal data set as the underlying data used to reach the conclusions drawn in the manuscript and any additional data required to replicate the reported study findings in their entirety. All PLOS journals require that the minimal data set be made fully available. For more information about our data policy, please see <http://journals.plos.org/plosone/s/data-availability>.

It already stated in the Methods section at the data were provided in the Supplementary material. Both reviewers noted this.

Upon re-submitting your revised manuscript, please upload your study’s minimal underlying data set as either Supporting Information files or to a stable, public repository and include the relevant URLs, DOIs, or accession numbers within your revised cover letter. For a list of acceptable repositories, please see http://journals.plos.org/plosone/s/data-availability#loc-recommended-repositories. Any potentially identifying patient information must be fully anonymized.

Important: If there are ethical or legal restrictions to sharing your data publicly, please explain these restrictions in detail. Please see our guidelines for more information on what we consider unacceptable restrictions to publicly sharing data: http://journals.plos.org/plosone/s/data-availability#loc-unacceptable-data-access-restrictions. Note that it is not acceptable for the authors to be the sole named individuals responsible for ensuring data access.

We will update your Data Availability statement to reflect the information you provide in your cover letter.

 This is now done.

4. Thank you for stating the following in the Financial Disclosure section:

The authors received no specific funding for this work.

We note that one or more of the authors are employed by a commercial company: Tacit London Ltd.

Tacit is the wholly owned company of the first author: it does not employ her. The Acknowledgments have been revised as requested.

a) Please provide an amended Funding Statement declaring this commercial affiliation, as well as a statement regarding the Role of Funders in your study. If the funding organization did not play a role in the study design, data collection and analysis, decision to publish, or preparation of the manuscript and only provided financial support in the form of authors' salaries and/or research materials, please review your statements relating to the author contributions, and ensure you have specifically and accurately indicated the role(s) that these authors had in your study. You can update author roles in the Author Contributions section of the online submission form.

Please also include the following statement within your amended Funding Statement.

“The funder provided support in the form of salaries for authors [insert relevant initials], but did not have any additional role in the study design, data collection and analysis, decision to publish, or preparation of the manuscript. The specific roles of these authors are articulated in the ‘author contributions’ section.”

If your commercial affiliation did play a role in your study, please state and explain this role within your updated Funding Statement.

This is now done.

b) Please also provide an updated Competing Interests Statement declaring this commercial affiliation along with any other relevant declarations relating to employment, consultancy, patents, products in development, or marketed products, etc.

Within your Competing Interests Statement, please confirm that this commercial affiliation does not alter your adherence to all PLOS ONE policies on sharing data and materials by including the following statement: "This does not alter our adherence to  PLOS ONE policies on sharing data and materials.” (as detailed online in our guide for authors <http://journals.plos.org/plosone/s/competing-interests>) . If this adherence statement is not accurate and  there are restrictions on sharing of data and/or materials, please state these. Please note that we cannot proceed with consideration of your article until this information has been declared.

Please include both an updated Funding Statement and Competing Interests Statement in your cover letter. We will change the online submission form on your behalf.

Now updated

=====================

Reviewers' comments:

Reviewer's Responses to Questions

**Comments to the Author**

1. Is the manuscript technically sound, and do the data support the conclusions?

The manuscript must describe a technically sound piece of scientific research with data that supports the conclusions. Experiments must have been conducted rigorously, with appropriate controls, replication, and sample sizes. The conclusions must be drawn appropriately based on the data presented.

Reviewer #1: Yes

Reviewer #2: Partly

2. Has the statistical analysis been performed appropriately and rigorously?

Reviewer #1: Yes

Reviewer #2: Yes

3. Have the authors made all data underlying the findings in their manuscript fully available?

The [PLOS Data policy](http://www.plosone.org/static/policies.action#sharing) requires authors to make all data underlying the findings described in their manuscript fully available without restriction, with rare exception (please refer to the Data Availability Statement in the manuscript PDF file). The data should be provided as part of the manuscript or its supporting information, or deposited to a public repository. For example, in addition to summary statistics, the data points behind means, medians and variance measures should be available. If there are restrictions on publicly sharing data—e.g. participant privacy or use of data from a third party—those must be specified.

Reviewer #1: Yes

Reviewer #2: Yes

4. Is the manuscript presented in an intelligible fashion and written in standard English?

PLOS ONE does not copyedit accepted manuscripts, so the language in submitted articles must be clear, correct, and unambiguous. Any typographical or grammatical errors should be corrected at revision, so please note any specific errors here.

Reviewer #1: Yes

Reviewer #2: Yes

5. Review Comments to the Author

Please use the space provided to explain your answers to the questions above. You may also include additional comments for the author, including concerns about dual publication, research ethics, or publication ethics. (Please upload your review as an attachment if it exceeds 20,000 characters)

Reviewer #1: In this manuscript, authors analyzed the fractal structure of communities of practice and revealed the relation between the size of CoP and the administrative structures. This work is of great importance and interests. And the manuscript can be improved if the following comments and suggestions can be considered in the revision.

1 In the introduction, it is better to provide clear and sufficient contribution to readers.

We have added additional material to the Introduction to address this point.

2 Some writing errors should be corrected. For example, in page 6, “SPPS” should be “SPSS”.

corrected

3 More clarity is required on what exactly the F-ratio and Silhouette index.
detail added to Methods section

4 In figure 4, there is a phase transition from the possibility of democracy to management by a small team of either 1-2 or 5-10 individuals. Why there are two types of management team size? Is there any intuitive explanation?

Thank you for the suggestion. We have now added some comments on this to the Discussion.

5 While the discussion section seems adequate, I suggest to add more to the application of results in organization management. I suppose the analysis in the context of business and administrative structures is a major contribution, some implications for business application would be really beneficial.

We have added some additional material to the Discussion to address this interesting point.

Reviewer #2: The paper analyses the size of community of practises. The main goal is to understand if natural groupings exist, giving rise to regular structures in social groupings, as it was demonstrated consistently in the literature both for offline and online communication means and ways of meeting.

To this end, authors collect a survey of around 100 individuals, who self report the sizes of the CoP they belong to. The paper provides (i) a cluster analysis to identify common sizes of communities; (ii) an analysis of the number of leaders that emerge as a function of the community size.

The paper derives the following main results: (i) well-formed clusters exist in group sizes. Specifically, 6 clusters can be found, and the sizes of typical human group sizes previously found in the literature are good fits for those sizes. (ii) communities cannot grow using an egalitarian way of management (i.e., without a management structure) above around 40 members, which is a size similar to those found in “non-layered” hunter-gatherer societies.

The paper provides interesting results on a less explored type of human social networks, with respect to existing literature. To the best of the reviewer’s knowledge, the structure of CoP was never analysed before.
The analysis of the collected result is methodologically sound, and the provided results in general back up well the claims about the existence of typical sizes for CoP communities, and the “scalability” properties of managing such groups.

One point, in this reviewer’s opinion, needs much better discussion, and maybe to gather additional data. Specifically, in the existing literature, the grouping of personal networks are normally considered from the ego standpoint, not from the entire community standpoint. Even more importantly, the sizes of the clusters typically observed in literature refer to individual relationships between an ego and their social ties. The same holds also for the frequency of interactions. Conceptually, making the analogy between groups in individual ego networks and entire communities is not straightforward, and would need to be better justified.

In fact, the literature does show that the layered structure of ego networks and of social communities is identical. However, we have added further comment on this to the Introduction to make the justification clearer.

Another point to be at least discussed in the paper is whether there exists an internal structure inside the observed communities. For example, do the large communities present internal clusters of individuals interacting at higher frequencies? Can layered structures be observed (as it is the case in most of the related literature authors consider)?

A good point. In fact, we have already commented on this point in respect of the need for a two-tier structure (organising committee + members in groups with >50 vs members only in small groups). We have added additional comments to the Discussion.

Finally, in this reviewer’s opinion, two aspects of the methodology should be described in more detail. Specifically, authors should explain more in detail the methodology behind the analysis of Figure 2c and Table 1, which are both described in a rather cursory way.

Detail now added to Methods.
